# The Calorie and Nutrient Density of More- Versus Less-Processed Packaged Food and Beverage Products in the Canadian Food Supply

**DOI:** 10.3390/nu11112782

**Published:** 2019-11-15

**Authors:** Laura Vergeer, Paige Veira, Jodi T. Bernstein, Madyson Weippert, Mary R. L’Abbé

**Affiliations:** Department of Nutritional Sciences, Faculty of Medicine, University of Toronto, Toronto, ON M5S 1A8, Canada; laura.vergeer@mail.utoronto.ca (L.V.); p.veira@mail.utoronto.ca (P.V.); jodi.bernstein@mail.utoronto.ca (J.T.B.); madyson.weippert@mail.utoronto.ca (M.W.)

**Keywords:** food processing, NOVA, nutritional quality, food supply

## Abstract

The association between the degree of processing and healthfulness of foods remains unclear. Most evidence of this relationship is based on dietary intake surveys rather than individual products and varies depending on the food processing classification system used. This study aimed to compare the nutritional quality of more- versus less-processed packaged foods and beverages in Canada, using a large, branded food database and two processing classification systems. Nutritional information for products (*n* = 17,269) was sourced from the University of Toronto FLIP 2017 database. Products were categorized using the NOVA and Poti et al. processing classification systems. Calories, sodium, saturated fat, total and free sugars, fibre and protein per 100 g (or mL) were examined by processing category using descriptive statistics and linear regression. Overall, the most-processed products under both systems were more likely to be lower in protein, and higher in total and free sugars, compared with less-processed foods (*p* < 0.05); the direction and strength of the association between other nutrients/components and level of processing were less consistent. These findings demonstrate that calorie- and nutrient-dense foods exist across different levels of processing, suggesting that food choices and dietary recommendations should be based primarily on energy or nutrient density rather than processing classification.

## 1. Introduction

Consistent with global trends, the Canadian food supply has become increasingly dominated by packaged, pre-prepared and highly processed food and beverage products [1,2,3]. Throughout the past century, advancements in food manufacturing technologies and distribution systems have improved the safety, shelf-life, taste and economies of scale of processed foods [4,5,6,7,8], contributing to their proliferation within national marketplaces [7,9,10]. Since the late 1930s, food purchases in Canada have been shifting from unprocessed or minimally processed foods toward ultra-processed alternatives [2]. As a result, ultra-processed products are estimated to constitute approximately half of Canadians’ daily energy intakes, on average [3].

There is some evidence to suggest that food processing may be related to diet quality and health [9,11,12,13,14,15,16,17]. Studies of national dietary intake surveys using generic food composition databases have shown a relationship between ultra-processed food consumption and less healthy diets in Canada [3,11], the United States [12], Brazil [17,18], France [15] and the United Kingdom [19], among others. In Canada, greater dietary shares of ultra-processed products have been associated with higher intakes of calories, carbohydrates, free sugars, total and saturated fats, and lower intakes of protein, fibre and several micronutrients [3]. Research has also found a positive relationship between greater consumption of ultra-processed foods and risk factors for disease, such as high BMI [20,21], hypertension [22], metabolic syndrome [23], altered lipid profiles [24], and an elevated breast cancer risk [25]. In light of this evidence, Canada’s recently updated Food Guide recommends limiting consumption of processed foods—especially those considered ultra-processed—and, when choosing these foods, eating them less often and in small amounts [26].

Conclusions about the nutritional quality of packaged foods and beverage products in relation to their degree of processing are, however, inconsistent. Studies have demonstrated that not all highly processed foods are of lower nutritional quality, leading some researchers to suggest that processed foods play an important role in the diet and that all levels of processing contribute both nutrients to encourage and to limit [27,28,29,30,31,32]. Furthermore, while the association of food processing with dietary intakes and disease risk factors has been well-documented [11,12,15,17,19], fewer studies have provided comprehensive comparisons of the nutritional composition of more- and less-processed individual branded food products in national food supplies, which is important in order to understand the degree to which ultra-processed foods may promote poor diet quality and health. Studies using large, branded food datasets in the United States, Australia and New Zealand have shown that more-processed foods are not necessarily higher in calories and negative nutrients or less aligned with national dietary guidelines than less-processed alternatives [13,29,30]. These studies were, however, limited to the application of single food processing classification systems, which have been shown to have important implications for the interpretation of research findings [33,34].

Overall, research concerning the relationship between the level of processing and nutritional quality of foods has generated discrepant findings. Few studies have investigated the healthfulness of processed foods using product-specific descriptions, nutritional information and ingredient lists, which are important to ensure accuracy of processing classifications and nutritional composition [11,13]. Differences between food processing classification systems have also contributed to varying conclusions about how processing relates to the healthfulness of foods and diets. The purpose of the present study was, therefore, to compare the nutritional quality of more- versus less-processed packaged food and beverage products in Canada, using a large, branded food dataset and two food processing classification systems.

## 2. Methods

### 2.1. Food Composition Data

Food composition information for products was sourced from the University of Toronto Food Label Information Program (FLIP) 2017, which is described elsewhere [35,36]. In summary, FLIP 2017 is a database of packaged food and beverage product labels collected from an outlet of each of three major Canadian grocery chains (Loblaws, Metro and Sobeys) in the Greater Toronto Area in the summer of 2017. It includes information such as a product’s Nutrition Facts table (NFt), ingredient list and photos of all sides of the packaging, among other things. Products in FLIP 2017 were classified according to the major (*n* = 24) and minor (*n* = 153) food categories defined in Health Canada’s Table of Reference Amounts for Food (TRA) [37]. In addition to nutritional information per serving size (as displayed on the NFt), calorie and nutrient contents of products were also determined per either 100 g or 100 mL for consistency within a food category. For products requiring preparation prior to consumption (e.g., condensed soups, cake mixes, beverage powders), their nutritional composition was also calculated according to the “as prepared” form, based on instructions listed on the product packaging.

### 2.2. Products Included in the Sample

Infant and toddler foods (*n* = 229), meal replacements and nutritional supplements (*n* = 82), non-alcoholic drink mixers (*n* = 20) and other products missing one or more of the nutrients of interest (*n* = 71) were excluded. Products in FLIP 2017 that were collected in multiple package sizes but had identical nutritional composition (according to the NFt and ingredients list) were examined only once; all flavours or varieties of a product were included. The final analytic sample consisted of 17,269 unique products.

### 2.3. Food Processing Classification Systems

In the absence of a consensus on the best framework for characterizing the processing level of foods in North America [27], products in FLIP 2017 were categorized according to two food processing classification systems: the NOVA system and an unnamed classification system created by Jennifer M. Poti et al. [13,38]. The NOVA system was developed by Carlos Monteiro and colleagues at the University of Sāo Paulo in 2009 and has since been strengthened through testing and consultation in several countries [39,40]. NOVA is recognized and applied by international bodies such as the World Health Organization (WHO) and the Food and Agricultural Organization of the United Nations, and it has been used in numerous studies on food purchasing and consumption throughout the world (27, 71). Poti et al. have since developed a more detailed food processing classification system guided by NOVA but adapted to reflect the varying degree to which foods are processed in the North American food supply [13]. This system has been used to examine the relationship between the extent of processing and nutritional quality of foods purchased and consumed in the United States, and whether front-of-package nutrition references on foods in Canada are associated with food processing [13,33,34]. Both the NOVA and Poti et al. systems distinguish between foods in terms of the nature, purpose and degree of processing [13,38].

NOVA classifies foods into one of four categories: (1) “unprocessed or minimally processed foods”; (2) “processed culinary ingredients”; (3) “processed foods”; and (4) “ultra-processed food and drink products” [38]. The Poti et al. system has seven categories, consisting of 4 major categories and 3 subcategories: (1) “unprocessed/minimally processed”; (2) “basic processed” (subcategories: “basic processed ingredients” and “processed for basic preservation”); (3) “moderately processed” (subcategories: “moderately processed for flavour” and “moderately processed grain products”); and (4) “highly processed” (subcategories: “highly processed ingredients” and “highly processed stand-alone”) [13]. Definitions and example foods for each category of the NOVA and Poti et al. systems are provided in Table S1. Classification of the entire sample of products according to the two systems was checked twice by the first author (L.V.) and a second researcher (P.V.) independently categorized a random 20% of the analytic sample using both the NOVA and Poti et al. systems. Inter-rater reliability was calculated using weighted Cohen’s Kappa, which found almost perfect agreement (kappa = 0.84 for NOVA; kappa = 0.85 for Poti et al.) [41].

### 2.4. Assessment of The Nutritional Quality of Products

The nutritional quality of products was evaluated in terms of their calorie, sodium, saturated fat, total sugars, free sugars, fibre and protein content per 100 g (or 100 mL). These nutrients and components were selected based on their inclusion in dietary guidelines and initiatives of Health Canada, the WHO and others [42,43]. They also form the basis of several government-endorsed nutrient profiling models that have been globally applied and validated, such as the Nutrient Profiling Scoring Criterion [44], the Health Star Rating system [45], Nutri-Score [15] and the UK Ofcom Nutrient Profile Model [46]. Free sugar contents were estimated using an algorithm developed by Bernstein et al., which is described elsewhere [35]. Both total and free sugars were examined as free sugars could not be estimated for products that displayed an NFt but not an ingredients list (e.g., baked goods or meals prepared in-store at Loblaws or Sobeys; *n* = 433). The unit of assessment (g or mL) was based on that for the TRA minor food category to which the product was assigned [37]. To facilitate comparisons between products within food categories, “as prepared” calorie and nutrient contents were used for beverage powders, baking and dessert mixes, concentrated sauces and gravies, condensed soups and combination dishes requiring preparation (e.g., boxed macaroni and cheese); “as sold” values were assessed for all other products.

### 2.5. Statistical Analysis

The number and proportion of products in each category of the NOVA and Poti et al. systems were calculated for the total sample and by TRA major food category. Calorie, sodium, saturated fat, total sugar, free sugar, fibre and protein amounts per 100 g (or 100 mL) across each level of processing were examined in terms of means, standard deviations and quartiles (minimum, 25th, median, 75th, maximum). Linear regression models were used to examine the association between level of processing and calories, sodium, saturated fat, total sugars, free sugars, fibre or protein per 100 g (or 100 mL). Separate models were constructed for each nutrient or component (outcome) and processing classification system (predictor: NOVA or Poti et al. category). In the regression analyses, the Poti et al. system was examined at the level of its four major categories: “unprocessed/minimally processed”; “basic processed”; “moderately processed”; and “highly processed”. All models adjusted for food category (as defined in Health Canada’s Table of Reference Amounts for Food) [37]. Robust sandwich variance estimators for linear regression were used to estimate 95% CIs and *p*-values in the presence of heteroscedasticity. Statistical significance was defined as *p* < 0.05. Analyses were completed using RStudio (version 1.1.456, RStudio Inc., Boston, MA, USA).

## 3. Results

### 3.1. Food Processing Classification

Results of the classification of products by level of processing according to the NOVA and Poti et al. systems are presented in Table 1, overall and by food category. Most packaged food and beverage products were classified as ultra-processed or highly processed according to the NOVA (73.5%) and Poti et al. (68.3%) systems, respectively. Fewer products were considered unprocessed or minimally processed (12.7%), processed (10.6%) or a processed culinary ingredient (3.1%) under NOVA. Based on the Poti et al. system, 52.2% were highly processed stand-alone products, 13.1% were highly processed ingredients, 16.3% were moderately processed (including 15.9% for flavour and 0.4% as grain products), 9.1% were basic processed (5.7% for basic preservation and 3.4% as ingredients) and 6.3% were unprocessed/minimally processed. All combination dishes, desserts, dessert toppings and fillings, and soups were considered ultra- or highly processed (100.0% for all); the food category with the fewest ultra- or highly processed products was legumes (2.7%). Eggs and egg substitutes had the greatest proportion of unprocessed or minimally processed foods according to both systems (85.2% for NOVA; 72.1% for Poti et al.). Notably, cereal and grain products were distributed throughout all four categories of the NOVA system and seven categories of the Poti et al. system.

### 3.2. Calorie and Nutrient Amounts Per 100 g (or 100 mL) by Extent of Processing

Distributions of calories, sodium, saturated fat, total sugars, free sugars, fibre and protein per 100 g (or 100 mL) for products in each category of the NOVA and Poti et al. food processing classification systems are presented for the overall sample in Table 2. Results of the linear regression analyses, adjusted for food category, are summarized below and reported in Table 3. Median amounts (and interquartile ranges) of the nutrients and components of interest in each NOVA and Poti et al. processing category per 100 g (or 100 mL) are shown by food category in Table 4. Discrepancies between trends in calorie or nutrient densities across processing categories within the total sample (Table 2) and the adjusted linear regression results (Table 3) highlight the variation in this relationship by food category.

#### 3.2.1. Calories

Within the total sample, mean and median calories in ultra-processed products were higher than for processed and unprocessed/minimally processed foods but lower than for processed culinary ingredients, according to the NOVA system (Table 2). The linear regression model adjusted for food category did, however, indicate that unprocessed/minimally processed (β = 16.51; *p* < 0.001) and processed foods (β = 13.14; *p* < 0.001) were more likely to be higher in calories than ultra-processed products (Table 3). Under the Poti et al. system, mean and median calories in highly processed ingredients and stand-alone foods in the overall sample were higher than those of unprocessed/minimally processed foods but comparable or lower than those of other processing categories. Similar to NOVA, according to the adjusted linear regression model, unprocessed/minimally processed (β = 13.24; *p* < 0.001), basic processed (β = 20.35; *p* < 0.001) and moderately processed foods (β = 6.78; *p* = 0.004) were more likely to be higher in calories per 100 g (or 100 mL), compared with highly processed products, based on the Poti et al. system (Table 3). For several food categories, the most-processed products (under both systems) had comparable or lower median numbers of calories than products processed to a lesser degree, such as: eggs and egg substitutes; fats and oils; fruit and fruit juices; legumes; and nuts and seeds (Table 4).

#### 3.2.2. Sodium

Under NOVA, overall median sodium contents of ultra-processed products were higher than those of unprocessed/minimally processed foods and processed culinary ingredients, but lower than those of processed foods (Table 2). The mean sodium content of processed culinary ingredients was, however, considerably higher than that of all other NOVA categories, suggesting that some processed culinary ingredients were very sodium-dense (Table 2). Based on the adjusted linear regression model, unprocessed/minimally processed foods were more likely to be lower in sodium (β = −137.67; *p* < 0.001), while processed culinary ingredients were more likely to be higher in sodium per (β = 1071.13; *p* = 0.002), compared with ultra-processed products (Table 3). According to the Poti et al. system, median sodium contents were greatest for highly processed foods and lowest for unprocessed/moderately processed or basic processed foods among the total sample (Table 2). However, similar to NOVA, mean sodium contents were considerably greater than the medians for basic processed ingredients and highly processed ingredients (and for all other Poti et al. categories besides moderately processed grain products; Table 2). Compared with highly processed products, unprocessed/minimally processed foods were more likely to be lower in sodium (β = −73.06; *p* = 0.03), while moderately processed foods were more likely to be higher in sodium (β = 115.52; *p* = 0.02; Table 3). Based on both the NOVA and Poti et al. systems, the most-processed bakery products and legumes had lesser or comparable median amounts of sodium per 100 g (or 100 mL) than products in lower processing categories (Table 4).

#### 3.2.3. Saturated Fat

Based on NOVA, median levels of saturated fat in ultra-processed products were lower than those of processed culinary ingredients among the overall sample, but higher than those of unprocessed/minimally processed foods and processed foods (Table 2). The mean saturated fat content of processed foods was slightly higher than for ultra-processed products. Compared with ultra-processed products, processed culinary ingredients (β = 5.61; *p* < 0.001) and processed foods (β = 0.80; *p* < 0.001) were more likely to be higher in saturated fat, when adjusted for food category (Table 3). Under the Poti et al. system, median saturated fat contents of highly processed products among the total sample were greater than those of all other categories; however, a less consistent pattern was observed among mean saturated fat amounts (Table 2). Compared with highly processed products, unprocessed/minimally processed (β = 0.80; *p* < 0.001), basic processed (β = 1.77; *p* < 0.001) and moderately processed foods (β = 1.06; *p* < 0.001) were more likely to be higher in saturated fat (Table 3), when adjusted for food category. According to both the NOVA and Poti et al. systems, ultra- or highly processed eggs and egg substitutes, fats and oils, marine and fresh-water animals, legumes, miscellaneous products, and vegetables had similar or lower median saturated fat contents than products in lower processing categories (Table 4).

#### 3.2.4. Total and Free Sugars

According to NOVA, overall median total and free sugars per 100 g (or 100 mL) in ultra-processed foods were greater than those of unprocessed/minimally processed foods, processed foods and culinary ingredients (Table 2). Compared with ultra-processed products, unprocessed/minimally processed (total sugars: β = −3.41; free sugars: β = −4.18; *p* < 0.001 for both) and processed foods (total sugars: β = −2.63; free sugars: β = −1.66; *p* < 0.001 for both) were more likely to be lower in total and free sugars (Table 3). Free sugars were also more likely to be lower in processed culinary ingredients than ultra-processed products (β = −2.68; *p* = 0.04; Table 3). Under the Poti et al. system, total and free sugar amounts were higher in highly processed products among the total sample than in unprocessed/minimally processed, basic processed and moderately processed foods (Table 2). Similarly, when adjusted for food category, unprocessed/minimally processed (total sugars: β = −2.53; free sugars: β = −4.01; *p* < 0.001 for both), basic processed (total sugars: β = −2.41; free sugars: β = −2.41; *p* < 0.001 for both) and moderately processed foods (total sugars: β = −1.22, *p* < 0.001; free sugars: β = −0.94, *p* = 0.001) were more likely to be lower in total and free sugars, compared with highly processed products (Table 3). Under both processing classification systems, mean total and free sugars contents were considerably higher for processed culinary ingredients (NOVA), and basic and highly processed ingredients (Poti et al. system), reflecting the high sugar density of many culinary ingredients (Table 2). Based on both systems, median total and free sugars contents of the most-processed products in the fruit and fruit juices, salads, snacks, and sugars and sweets categories were comparable or lower than those of products processed to lesser extents (Table 4).

#### 3.2.5. Fibre

Under NOVA, mean and median fibre contents of ultra-processed products among the total sample were comparable to those of foods in other processing categories for the total sample (Table 2). Compared with ultra-processed products, unprocessed/minimally processed foods were more likely to be higher in fibre (β = 1.13; *p* < 0.001), while processed foods were more likely to be lower in fibre (β = −0.67; *p* < 0.001), when adjusted for food category (Table 3). Based on the Poti et al. system, overall mean and median fibre amounts in highly processed products were highest for moderately processed grain products and similar among other processing categories (Table 2). The adjusted linear regression model indicated that unprocessed/minimally processed (β = 2.42; *p* < 0.001) and moderately processed foods (β = 0.50; *p* < 0.001) were more likely to be higher in fibre, compared with highly processed products (Table 3). Under both the NOVA and Poti et al. systems, the most-processed cereals and other grain products, potatoes, and salads had comparable or higher median fibre contents per 100 g (or 100 mL) compared to products in lower processing categories (Table 4).

#### 3.2.6. Protein

According to NOVA, average protein contents of ultra-processed foods per 100 g (or 100 mL) in the total sample were higher than those of processed culinary ingredients but less than those of unprocessed minimally processed and processed foods (Table 2). Compared with ultra-processed products, unprocessed/minimally processed (β = 2.39; *p* < 0.001) and processed foods (β = 1.77; *p* < 0.001) were more likely to be higher in protein (Table 3), when adjusted for food category. Based on the Poti et al. system, mean and median protein amounts were greatest for moderately processed grain products, followed by products processed for basic preservation, products moderately processed for flavour and highly processed stand-alone products (Table 2). The adjusted linear regression model indicated that unprocessed/minimally processed (β = 3.64; *p* < 0.001), basic processed (β = 1.32; *p* < 0.001) and moderately processed foods (β = 1.54; *p* < 0.001) were more likely to be higher in protein, compared with highly processed products (Table 3). Under both the NOVA and Poti et al. systems, the most-processed eggs and egg substitutes, miscellaneous products and potatoes had similar or greater median protein contents per 100 g/mL than those in lower processing categories (Table 4).

## 4. Discussion

This study provides a comprehensive evaluation of the nutritional quality of packaged foods and beverages in relation to their extent of processing, drawing on a highly representative dataset of branded products in the Canadian food supply. More than two-thirds of the sampled products were deemed ultra- or highly processed according to the NOVA and Poti et al. systems. This result is consistent with an earlier application of these systems to a Canadian sample of packaged foods and beverages [34]. Among the total sample, unprocessed or minimally processed foods contained comparable or more favourable average amounts of sodium, saturated fat, total and free sugars, fibre and protein per 100 g (or 100 mL) than products in all other processing categories. The relationship between calories and level of processing was less consistent, with considerable variation in this trend by food category. There was also less difference in nutrient densities between ultra- or highly processed products and processed foods or ingredients (i.e., “processed foods” or “processed culinary ingredients” under NOVA; “basic” or “moderately” processed foods or ingredients based on the Poti et al. system) in several food categories. Our findings indicate that calorie- and nutrient-dense foods exist across different levels of processing and it may not be reasonable to recommend avoidance of a particular food strictly because it is considered ultra- or highly processed.

Our results support previous research demonstrating that virtually all packaged food is processed to some extent, and products are not simply healthy or unhealthy based solely on whether they have been processed [13,21,27,28,31,33,47,48,49]. For several food categories, products processed to greater extents had similar or healthier calorie and/or nutrient densities than products in lower processing classifications. This result is consistent with a recent examination of the nutritional composition of 100 foods commonly consumed by American children, which found that nutrient concentrations were not strongly predictive of a food’s processing classification as defined by the NOVA and Poti et al. systems [33]. Furthermore, in our overall sample and within food categories, there were large ranges in calorie and nutrient densities among products in the same processing categories, demonstrating the variation in nutritional composition that exists between products processed to similar extents. In applying their classification system to processed food purchases of American households, Poti et al. also found wide variation in the saturated fat, sodium and total sugars content of more- and less-processed products within the same processing categories [13]. Findings from these studies suggest that food choices and dietary recommendations should be based primarily on nutrient density rather than level of processing, since products within processing categories differ widely in nutritional quality [13,31,32]. Observed ranges in the calorie and nutrient composition of products in the same processing categories also highlight opportunities for manufacturers to reformulate and improve the nutritional quality of many processed foods. Healthier processed foods offer many potential benefits, including greater affordability, convenience, enhanced safety, longer shelf-lives and fortification with essential micronutrients to help prevent deficiencies [10,50,51]. Avoiding processed foods of higher nutritional quality may have negative implications for diet quality, health and wellbeing [31,32].

The mechanism underlying the observed associations of highly processed foods with greater daily energy intakes and poor health outcomes remains unclear; however, research suggests this may be more related to the physical and structural characteristics of these foods than their nutritional composition. It has been proposed that ultra-processed products are typically engineered to be hyper-palatable and addictive, which may disrupt gut–brain signaling pathways that regulate satiety and appetite, and potentially contribute to overconsumption [52,53]. A recent randomized controlled feeding trial further supported the theory that the effects of processed foods extend beyond their calorie and nutrient density [21]. The study found that participants fed an ultra-processed diet (as defined by NOVA) for two weeks—matched to an unprocessed diet for presented calories, sugar, fat, sodium, fibre and macronutrients—consumed approximately 500 more calories per day than when eating an unprocessed diet and gained an average of ~1 kg of body weight during that period [21]. Although further research is needed to elucidate the mechanisms behind these differences, the contribution of highly processed foods to overconsumption and weight gain appears to be related to components or attributes other than just their nutritional composition [21,52,53,54]. Our results support this notion by demonstrating that many highly processed products retain equal or more favourable calorie and nutrient densities than foods in lower processing categories.

It may, therefore, be most critical to focus on the amounts, frequencies and combinations in which highly processed products are consumed, given that previous studies have associated greater dietary shares of these foods with poorer quality diets and adverse health outcomes [11,12,15,17,18,21,22,55]. In the present study, mean densities of calories, saturated fat, sodium and/or sugars in processed culinary ingredients (NOVA) and basic and highly processed ingredients (Poti et al. system) were higher than or similar to those of ultra-processed or highly processed products. There are, however, important considerations with interpreting these results. First, calories and nutrient amounts were examined per 100 g (or 100 mL), which is considerably larger than the typical serving size for ingredients. For example, Health Canada has set reference amounts for butter, oils, sugars, starches and other ingredients of ≤30 g or mL [37]. Thus, although processed culinary ingredients are often dense in calories and negative nutrients, they are consumed in relatively small amounts and are estimated to constitute only about 6% of Canadians’ daily energy intakes, on average [11]. Examining the nutritional quality of processed ingredients per serving may have suggested more favourable nutrient profiles for foods in these processing categories.

In classifying products based on their extent of processing, the NOVA and Poti et al. systems generated similar results overall. Notably, both systems effectively distinguished between the least- and most-processed foods, with unprocessed or minimally processed foods consistently containing more favourable average amounts of both positive and negative nutrients per 100 g (or 100 mL), compared with ultra-processed or highly processed products. These findings support current public health recommendations encouraging consumption of more unprocessed or minimally processed foods and fewer highly processed alternatives, such as Canada’s recently updated Food Guide [56]. Government policy initiatives to complement Canada’s Food Guide and indirectly reduce Canadians’ consumption of highly processed foods may be warranted, given that these products account for approximately half of Canadians’ average daily energy intakes, with particularly high intakes among children and adolescents, and First Nations adults living on-reserve [3,57]. Importantly, such interventions should account for the wide range in nutritional quality among processed and highly processed foods and beverages, as indicated by the results of this study. For example, mandatory interpretative front-of-package labelling may help consumers differentiate between healthier and less healthy processed foods by identifying those that are lower in nutrients of public health concern. In selecting foods and making dietary recommendations, it is also important to consider how the nutritional quality of a food may be impacted by processing-induced chemical contaminants, the substances that form in certain foods as a result of chemical modifications to their ingredients during processing (e.g., acrylamide, furan) [58]. Recommending avoidance or less frequent consumption of foods containing processing-induced chemical contaminants posing a potential health risk (e.g., French fries, potato chips and other fried foods containing acrylamide) may be one way to minimize dietary exposure to these substances [59,60]. Governments and other health authorities can also work with food manufacturers to develop and implement reduction strategies and monitoring programs to limit food processing-induced chemical contaminants in their products [60].

Despite their similarities, there are also important differences between the NOVA and Poti et al. food processing classification systems that may help to explain some of the observed trends in nutritional composition between processing levels. The main differences arise in how the two systems treat added sugars, salt or fat, and how they distinguish between whole- and refined-grains. For example, according to NOVA, flavoured milks and yogurts, unsweetened breakfast cereals, and packaged wholegrain breads are considered ultra-processed [38], whereas under the Poti et al. system, these products are deemed moderately processed [13]. This was reflected in our observation that, among cereals and grain products, median fibre content was higher for ultra-processed foods than other NOVA categories. NOVA has been criticized for its lack of consideration regarding the processing methods or technology used, focusing instead on the addition of sugar, salt or additives [31,32]. Furthermore, compared with other classification systems, NOVA is less able to account for variation in the extent of processing within food categories [31]. We found the Poti et al. system better distinguished between varying degrees of processing within several food categories, as evidenced by the distribution of these products across a greater number of processing classifications than under NOVA (e.g., cereals and other grain products, dairy products and substitutes, fruit and fruit juices, seafood, etc.). Another notable strength of the Poti et al. system compared with NOVA is the availability of a single document outlining clear definitions and examples for a wide variety of products (Supplemental Table 2 of [13]). Although NOVA is the most commonly and globally applied food processing classification system, products are not consistently categorized between studies [32], likely due to the ambiguity inherent in the system and available classification guidelines. Overall, our findings highlight the potential influence of the processing classification system on the perceived healthfulness of a product, and suggest that systems with a greater number of more precisely defined processing categories may prove most applicable to food supplies such as Canada’s that are dominated by processed foods.

A strength of this study is its use of a large and diverse dataset of packaged food and beverage products in Canada. Notably, access to branded data—including product-specific NFts and ingredient lists—helped to accurately assess the processing level and nutritional composition of foods, a common limitation of studies relying on national dietary intake surveys that use generic food composition databases [11]. This study is also strengthened by its application of two food processing classification systems, as differences between systems likely have critical implications for research findings [34,61]. Limitations of this work include that data for FLIP 2017 were collected from only three retail outlets in one major Canadian city at a single point in time and thus, did not capture all products in the Canadian food supply. Importantly, the FLIP 2017 database excludes products not required to display a Canadian NFt, such as fresh and unpackaged fruits, vegetables, meats and seafood that are typically considered unprocessed or minimally processed, the prevalence of which was, therefore, underestimated in our analysis. Additionally, this study’s evaluation of the nutritional quality of a product was limited to its composition of seven nutrients or components per 100 g (or 100 mL). Examination of a broader variety of nutrients and food components—and evaluating their amounts per serving size—may have yielded different results. Finally, this study did not examine the contribution of more- and less-processed foods to the diet, which is important to consider in making public health recommendations concerning the consumption of processed foods. Future research linking the branded FLIP database to dietary intake data from the Canadian Community Health Survey may enable a better understanding of how the nutritional composition of processed foods relates to Canadian intakes, diet quality and health.

## 5. Conclusions

More than two-thirds of packaged food and beverage products in Canada are considered ultra- or highly processed. While unprocessed or minimally processed foods typically have the best nutritional profile overall, many products deemed most-processed have similar or more favourable calorie or nutrient densities than those processed to lesser degrees and there is considerable variation in the nutritional composition of products in the same processing categories. Findings from this study suggest that in making food choices and providing dietary recommendations, it may be more important to focus on the energy or nutrient density of a food rather than its processing classification as calorie- and nutrient-dense foods exist across different levels of processing. This study also illustrates that the relationship between the processing level and calorie or nutrient density of a food varies based on the processing classification system used. Future studies examining how processing relates to the nutritional quality of individual branded foods and the frequency and amounts in which they are consumed by Canadians may be warranted.

## Figures and Tables

**Table 1:** The number and proportion of packaged food and beverage products in each category of the NOVA and Poti et al. food processing classification systems ^1^, overall and by food category.

	Total *n* ^3^	NOVA								Poti et al.													
Food Category ^2^		U/MP ^4^		PCI ^5^		P ^6^		UP ^7^		U/MP ^4^		BPI ^8^		PBP ^9^		MPF ^10^		MPG ^11^		HPI ^12^		HPS ^13^	
		*n*	%	*n*	%	*n*	%	*n*	%	*n*	%	*n*	%	*n*	%	*n*	%	*n*	%	*n*	%	*n*	%
OVERALL	17,269	2195	12.7	542	3.1	1833	10.6	12,699	73.5	1089	6.3	588	3.4	989	5.7	2746	15.9	67	0.4	2261	13.1	9529	55.2
A. Bakery products	2771	0	0.0	0	0.0	146	5.3	2625	94.7	0	0.0	0	0.0	0	0.0	0	0.0	51	1.8	0	0.0	2720	98.2
B. Beverages	840	101	12.0	1	0.1	6	0.7	732	87.1	82	9.8	20	2.4	4	0.5	26	3.1	0	0.0	1	0.1	707	84.2
C. Cereals and other grain products	1275	734	57.6	8	0.6	23	1.8	510	40.0	145	11.4	83	6.5	446	35.0	140	11.0	16	1.3	4	0.3	441	34.6
D. Dairy products and substitutes	1495	143	9.6	0	0.0	407	27.2	945	63.2	59	3.9	0	0.0	98	6.6	821	54.9	0	0.0	66	4.4	451	30.2
E. Desserts	679	0	0.0	0	0.0	0	0.0	679	100.0	0	0.0	0	0.0	0	0.0	0	0.0	0	0.0	0	0.0	679	100.0
F. Dessert toppings and fillings	94	0	0.0	0	0.0	0	0.0	94	100.0	0	0.0	0	0.0	0	0.0	0	0.0	0	0.0	94	100.0	0	0.0
G. Eggs and egg substitutes	61	52	85.2	0	0.0	0	0.0	9	14.8	44	72.1	8	13.1	0	0.0	0	0.0	0	0.0	0	0.0	9	14.8
H. Fats and oils	656	0	0.0	231	35.2	0	0.0	425	64.8	2	0.3	191	29.1	0	0.0	37	5.6	0	0.0	426	64.9	0	0.0
I. Marine and fresh water animals	446	57	12.8	0	0.0	202	45.3	187	41.9	57	12.8	0	0.0	13	2.9	189	42.4	0	0.0	0	0.0	187	41.9
J. Fruit and fruit juices	1056	543	51.4	0	0.0	213	20.2	300	28.4	155	14.7	138	13.1	293	27.7	196	18.6	0	0.0	0	0.0	274	25.9
K. Legumes	188	92	48.9	0	0.0	91	48.4	5	2.7	79	42.0	0	0.0	32	17.0	72	38.3	0	0.0	0	0.0	5	2.7
L. Meat, products and substitutes	960	31	3.2	0	0.0	93	9.7	836	87.1	30	3.1	0	0.0	0	0.0	130	13.5	0	0.0	0	0.0	800	83.3
M. Miscellaneous category	550	29	5.3	101	18.4	0	0.0	420	76.4	11	2.0	53	9.6	0	0.0	74	13.5	0	0.0	197	35.8	215	39.1
N. Combination dishes	1130	0	0.0	0	0.0	0	0.0	1130	100.0	0	0.0	0	0.0	0	0.0	0	0.0	0	0.0	0	0.0	1130	100.0
O. Nuts and seeds	255	148	58.0	48	18.8	0	0.0	59	23.1	143	56.1	0	0.0	43	16.9	48	18.8	0	0.0	0	0.0	21	8.2
P. Potatoes, sweet potatoes and yams	132	14	10.6	0	0.0	15	11.4	103	78.0	14	10.6	0	0.0	2	1.5	40	30.3	0	0.0	0	0.0	76	57.6
Q. Salads	129	2	1.6	0	0.0	14	10.9	113	87.6	2	1.6	0	0.0	0	0.0	1	0.8	0	0.0	0	0.0	126	97.7
R. Sauces/dips/gravies/condiments	1239	0	0.0	36	2.9	0	0.0	1203	97.1	0	0.0	0	0.0	0	0.0	0	0.0	0	0.0	1239	100.0	0	0.0
S. Snacks	861	10	1.2	0	0.0	132	15.3	719	83.5	10	1.2	0	0.0	0	0.0	359	41.7	0	0.0	0	0.0	492	57.1
T. Soups	480	0	0.0	0	0.0	0	0.0	480	100.0	0	0.0	0	0.0	0	0.0	0	0.0	0	0.0	0	0.0	480	100.0
U. Sugars and sweets	1106	6	0.5	117	10.6	0	0.0	983	88.9	43	3.9	69	6.2	0	0.0	179	16.2	0	0.0	219	19.8	596	53.9
V. Vegetables	866	233	26.9	0	0.0	491	56.7	142	16.4	213	24.6	26	3.0	58	6.7	434	50.1	0	0.0	15	1.7	120	13.9

^1^ Definitions and examples of products in the NOVA and Poti et al. processing categories are provided in Supplemental Table 1. ^2^ Food categories are defined in Health Canada’s Table of Reference Amounts for Food [37]. ^3^ Number of products, overall or by food category. ^4^ U/MP: unprocessed or minimally-processed products; ^5^ PCI: processed culinary ingredients. ^6^ P: processed foods. ^7^ UP: ultra-processed food and drink products. ^8^ BPI: basic processed ingredients. ^9^ PBP: processed for basic preservation. ^10^ MPF: moderately processed for flavour. ^11^ MPG: moderately processed grain products. ^12^ HPI: highly processed ingredients. ^13^ HPS: highly processed stand-alone.

**Table 2:** The distribution of calories, sodium, saturated fat, total sugars, free sugars, fibre and protein per 100 g (or 100 mL) in each category of the NOVA and Poti et al. food processing classification systems, presented for the total sample of packaged food and beverage products (*n* = 17,269) ^1^.

Nutritional Composition per 100 g/mL		NOVA				Poti et al.						
		U/MP ^2^	PCI ^3^	P ^4^	UP ^5^	U/MP ^2^	BPI ^6^	PBP ^7^	MPF ^8^	MPG ^9^	HPI ^10^	HPS ^11^
Calories (kcal)	Mean (SD)	215 (188)	521 (291)	219 (177)	254 (172)	226 (218)	391 (326)	216 (173)	247 (193)	320 (75)	228 (183)	260 (166)
	Min	0	0	0	0	0	0	0	0	200	0	0
	25th	48	300	64	100	41	48	48	80	250	80	110
	Median	138	600	186	240	132	360	167	200	316	200	255
	75th	356	800	350	400	360	800	356	376	367	333	400
	Max	760	1382	1060	1163	900	942	1382	1060	533	1163	670
Sodium (mg)	Mean (SD)	26 (83)	2013 (6203)	521 (744)	477 (1070)	34 (101)	550 (3839)	20 (65)	668 (2329)	288 (213)	1105 (2290)	360 (346)
	Min	0	0	0	0	0	0	0	0	0	0	0
	25th	0	0	100	73	0	0	0	40	33	224	95
	Median	5	0	365	304	6	0	4	230	300	500	296
	75th	24	13	679	533	38	8	14	600	460	933	492
	Max	2100	40,000	8796	33,000	2100	40,000	1067	36,000	667	33,000	3,378
Saturated fat (g)	Mean (SD)	1 (3)	12 (21)	5 (7)	4 (6)	2 (4)	9 (19)	1 (3)	5 (8)	1 (1)	3 (5)	4 (6)
	Min	0	0	0	0	0	0	0	0	0	0	0
	25th	0	0	0	0	0	0	0	0	0	0	0
	Median	0	5	1	2	0	0	0	1	0	0	2
	75th	1	15	9	5	2	10	0	7	1	3	6
	Max	57	100	48	94	60	100	57	94	5	50	50
Total sugars (g)	Mean (SD)	5 (10)	21 (36)	5 (13)	14 (18)	8 (19)	13 (29)	5 (5)	10 (19)	3 (5)	17 (23)	12 (15)
	Min	0	0	0	0	0	0	0	0	0	0	0
	25th	0	0	0	2	0	0	0	0	0	0	1
	Median	2	0	0	6	2	1	3	2	0	7	5
	75th	7	25	4	20	5	9	9	11	4	27	19
	Max	81	114	88	260	90	114	22	111	22	260	100
Free sugars (g) ^1^	Mean (SD)	2 (6)	19 (36)	2 (8)	12 (17)	3 (16)	13 (29)	2 (4)	7 (15)	1 (3)	15 (22)	11 (15)
	Min	0	0	0	0	0	0	0	0	0	0	0
	25th	0	0	0	0	0	0	0	0	0	0	0
	Median	0	0	0	4	0	0	0	0	0	4	3
	75th	0	0	0	16	0	9	3	7	0	21	15
	Max	81	114	87	143	90	114	16	111	17	143	100
Fibre (g)	Mean (SD)	4 (6)	2 (7)	2 (3)	2 (3)	6 (8)	2 (5)	2 (3)	2 (4)	9 (6)	1 (4)	2 (3)
	Min	0	0	0	0	0	0	0	0	0	0	0
	25th	0	0	0	0	0	0	0	0	6	0	0
	Median	2	0	1	1	2	0	2	0	8	0	1
	75th	6	0	3	3	8	0	4	4	13	2	3
	Max	100	90	40	43	100	50	40	40	27	90	43
Protein (g)	Mean (SD)	8 (8)	4 (9)	12 (10)	6 (7)	9 (9)	3 (7)	7 (7)	10 (10)	10 (3)	2 (3)	7 (7)
	Min	0	0	0	0	0	0	0	0	5	0	0
	25th	1	0	2	1	1	0	1	1	9	0	3
	Median	7	0	9	5	5	0	7	6	10	1	6
	75th	13	1	21	9	16	1	13	18	12	3	10
	Max	77	67	64	50	57	67	77	64	20	33	50

^1^ Distributions of free sugars exclude products without an ingredients list as free sugars could not be estimated (*n* = 433). Free sugars were estimated for the remaining 16,836 products with an ingredients list. ^2^ U/MP: unprocessed or minimally-processed products; ^3^ PCI: processed culinary ingredients. ^4^ P: processed foods. ^5^ UP: ultra-processed food and drink products. ^6^ BPI: basic processed ingredients. ^7^ PBP: processed for basic preservation. ^8^ MPF: moderately processed for flavour. ^9^ MPG: moderately processed grain products. ^10^ HPI: highly processed ingredients. ^11^ HPS: highly processed stand-alone.

**Table 3:** Results of the linear regression analyses examining calories, sodium, saturated fat, total sugars, free sugars, fibre and protein per 100 g (or 100 mL) by level of processing according to both the NOVA and Poti et al. classification systems (*n* = 17,269) ^1,2,3^.

		NOVA				Poti et al.			
Nutrient/Component		Unprocessed/Minimally Processed	Processed Culinary Ingredients	Processed Foods	Ultra-Processed ^3^	Unprocessed/Minimally Processed	Basic Processed	Moderately Processed	Highly Processed ^3^
Calories (kcal)	β	16.51	−3.87	13.14	Reference	13.24	20.35	6.78	Reference
	95% CI	12.27, 20.76	−23.14, 15.39	8.57–17.72	-	6.45, 20.02	14.55, 26.15	2.15, 11.42	-
	*p*-value	<0.001	0.69	<0.001	-	<0.001	<0.001	0.004	-
Sodium (mg)	β	−137.67	1071.13	10.16	Reference	−73.06	121.08	115.52	Reference
	95% CI	−156.89, −118.44	401.21, 1741.06	−27.81, 48.14	-	−140.94, −5.19	−44.85, 287.01	16.62, 214.41	-
	*p*-value	<0.001	0.002	0.60	-	0.03	0.15	0.02	-
Saturated fat (g)	β	0.16	5.61	0.80	Reference	0.80	1.77	1.06	Reference
	95% CI	−0.03, 0.35	4.02, 7.21	0.57, 1.02	-	0.51, 1.09	1.25, 2.30	0.80, 1.32	-
	*p*-value	0.10	<0.001	<0.001	-	<0.001	<0.001	<0.001	-
Total sugars (g)	β	−3.41	−0.15	−2.63	Reference	−2.53	−2.41	−1.22	Reference
	95% CI	−3.85, −2.97	−2.88, 2.59	−3.12, −2.13	-	−3.27, −1.78	−3.14, −1.68	−1.86, −0.59	-
	*p*-value	<0.001	0.92	<0.001	-	<0.001	<0.001	<0.001	-
Free sugars (g) ^2^	β	−4.18	−2.68	−1.66	Reference	−4.01	−2.41	−0.94	Reference
	95% CI	−4.58, −3.78	−5.26, −0.08	−2.18, −1.14	-	−4.75, −3.27	−3.12, −1.70	−1.51, −0.36	-
	*p*-value	<0.001	0.04	<0.001	-	<0.001	<0.001	0.001	-
Fibre (g)	β	1.13	−0.15	−0.67	Reference	2.42	0.19	0.50	Reference
	95% CI	0.91, 1.34	−0.87, 0.57	−0.85, −0.49	-	2.04, 2.81	−0.08, 0.47	0.30, 0.69	-
	*p*-value	<0.001	0.69	<0.001	-	<0.001	0.17	<0.001	-
Protein (g)	β	2.39	−0.28	1.77	Reference	3.64	1.32	1.54	Reference
	95% CI	2.16, 2.62	−1.03, 0.46	1.44, 2.10	-	3.28, 3.99	1.06, 1.58	1.27, 1.81	-
	*p*-value	<0.001	0.47	<0.001	-	<0.001	<0.001	<0.001	-

^1^ All models were adjusted for food category (defined in Health Canada’s Table of Reference Amounts for Food) [37]. ^2^ Distributions of free sugars exclude products without an ingredients list as free sugars could not be estimated (*n* = 433). Free sugars were estimated for the remaining 16,836 products with an ingredients list. ^3^ Indicates the highest level of processing for each classification system and the reference category.

**Table 4:** Median amounts of calories, sodium, saturated fat, total sugars, free sugars, fibre and protein per 100 g (or 100 mL) for products in each processing classification of the NOVA and Poti et al. systems, presented by food category ^1^.

			NOVA						Poti et al.				
Food Category ^1^	Nutritional Composition per 100 g/mL		U/MP ^2^	PCI ^3^	P ^4^	UP ^5^	U/MP ^2^	BPI ^6^	PBP ^7^	MPF ^8^	MPG ^9^	HPI ^10^	HPS ^11^
A. Bakery products	*n*		0	0	146	2625	0	0	0	0	51	0	2720
	Median (IQR) ^12^	Calories (kcal)	-	-	264 (59)	400 (151)	-	-	-	-	280 (122)	-	400 (153)
		Sodium (mg)	-	-	500 (190)	338 (269)	-	-	-	-	400 (277)	-	347 (268)
		Saturated fat (g)	-	-	1 (1)	4 (7)	-	-	-	-	0 (1)	-	3 (7)
		Total sugars (g) ^13^	-	-	2 (4)	18 (26)	-	-	-	-	0 (3)	-	17 (25)
		Free sugars (g)	-	-	0 (0)	12 (25)	-	-	-	-	0 (0)	-	12 (25)
		Fibre (g)	-	-	2 (2)	3 (4)	-	-	-	-	8 (7)	-	3 (4)
		Protein (g)	-	-	9 (2)	7 (5)	-	-		-	10 (2)	-	7 (5)
B. Beverages	*n*		101	1	6	732	82	20	4	26	0	1	707
	Median (IQR) ^12^	Calories (kcal)	0 (0)	0 (0)	0 (0)	22 (41)	0 (0)	18 (19)	0 (5)	26 (22)	-	0 (0)	22 (42)
		Sodium (mg)	0 (3)	0 (0)	1 (3)	6 (18)	0 (1)	20 (14)	7 (11)	4 (14)	-	0 (0)	6 (18)
		Saturated fat (g)	0 (0)	0 (0)	0 (0)	0 (0)	0 (0)	0 (0)	0 (0)	0 (0)	-	0 (0)	0 (0)
		Total sugars (g) ^13^	0 (0)	0 (0)	0 (0)	5 (10)	0 (0)	3 (4)	0 (1)	5 (7)	-	0 (0)	5 (10)
		Free sugars (g)	0 (0)	0 (0)	0 (0)	5 (10)	0 (0)	3 (4)	0 (1)	5 (7)	-	0 (0)	5 (10)
		Fibre (g)	0 (0)	0 (0)	0 (0)	0 (0)	0 (0)	0 (0)	0 (0)	0 (0)	-	0 (0)	0 (0)
		Protein (g)	0 (0)	0 (0)	0 (0)	0 (0)	0 (0)	0 (0)	0 (0)	0 (0)	-	0 (0)	0 (0)
C. Cereals and other grain products	*n*		734	8	23	510	145	83	446	140	16	4	441
	Median (IQR) ^12^	Calories (kcal)	356 (14)	342 (43)	357 (63)	367 (89)	375 (38)	358 (17)	356 (12)	365 (24)	362 (26)	310 (119)	367 (113)
		Sodium (mg)	0 (10)	0 (0)	62 (276)	322 (327)	0 (11)	0 (2)	0 (6)	65 (334)	0 (33)	1083 (2347)	322 (362)
		Saturated fat (g)	0 (0)	0 (0)	0 (2)	1 (2)	1 (1)	0 (0)	0 (0)	1 (1)	0 (2)	0 (0)	1 (2)
		Total sugars (g) ^13^	2 (2)	0 (0)	1 (5)	15 (21)	0 (2)	1 (3)	2 (2)	4 (19)	0 (9)	0 (7)	11 (20)
		Free sugars (g)	0 (0)	0 (0)	0 (0)	11 (19)	0 (0)	0 (0)	0 (0)	0 (19)	0 (0)	0 (7)	7 (18)
		Fibre (g)	4 (7)	0 (19)	4 (14)	6 (7)	10 (7)	8 (6)	4 (2)	6 (6)	12 (5)	39 (81)	5 (6)
		Protein (g)	13 (5)	0 (0)	12 (5)	9 (4)	13 (6)	13 (7)	13 (6)	12 (4)	12 (2)	0 (0)	9 (4)
D. Dairy products and substitutes	*n*		143	0	407	945	59	0	98	821	0	66	451
	Median (IQR) ^12^	Calories (kcal)	57 (22)	-	367 (100)	104 (211)	52 (22)	-	67 (44)	147 (281)	-	232 (142)	267 (257)
		Sodium (mg)	48 (11)	-	667 (267)	63 (589)	46 (9)	-	50 (24)	72 (622)	-	33 (75)	667 (539)
		Saturated fat (g)	1 (2)	-	17 (7)	2 (12)	1 (1)	-	1 (3)	5 (16)	-	7 (10)	12 (15)
		Total sugars (g) ^13^	4 (2)	-	0 (0)	7 (11)	5 (0)	-	3 (3)	3 (11)	-	7 (27)	0 (5)
		Free sugars (g)	0 (0)	-	0 (0)	2 (8)	0 (0)	-	0 (0)	0 (8)	-	4 (29)	0 (0)
		Fibre (g)	0 (0)	-	0 (0)	0 (0)	0 (0)	-	0 (0)	0 (0)	-	0 (0)	0 (0)
		Protein (g)	4 (2)	-	23 (7)	6 (10)	4 (0)	-	4 (3)	9 (19)	-	1 (1)	14 (17)
E. Desserts	*n*		0	0	0	679	0	0	0	0	0	0	679
	Median (IQR) ^12^	Calories (kcal)	-	-	-	120 (73)	-	-	-	-	-	-	120 (73)
		Sodium (mg)	-	-	-	52 (47)	-	-	-	-	-	-	52 (47)
		Saturated fat (g)	-	-	-	3 (4)	-	-	-	-	-	-	3 (4)
		Total sugars (g) ^13^	-	-	-	14 (6)	-	-	-	-	-	-	14 (6)
		Free sugars (g)	-	-	-	11 (7)	-	-	-	-	-	-	11 (7)
		Fibre (g)	-	-	-	0 (1)	-	-	-	-	-	-	0 (1)
		Protein (g)	-	-	-	2 (2)	-	-	-	-	-	-	2 (2)
F. Dessert toppings and fillings	*n*		0	0	0	94	0	0	0	0	0	94	0
	Median (IQR) ^12^	Calories (kcal)	-	-	-	326 (239)	-	-	-	-	-	326 (239)	-
		Sodium (mg)	-	-	-	96 (190)	-	-	-	-	-	96 (190)	-
		Saturated fat (g)	-	-	-	0 (4)	-	-	-	-	-	0 (4)	-
		Total sugars (g) ^13^	-	-	-	49 (34)	-	-	-	-	-	49 (34)	-
		Free sugars (g)	-	-	-	48 (43)	-	-	-	-	-	48 (43)	-
		Fibre (g)	-	-	-	0 (1)	-	-	-	-	-	0 (1)	-
		Protein (g)	-	-	-	0 (1)	-	-	-	-	-	0 (1)	-
G. Eggs and egg substitutes	*n*		52	0	0	9	44	8	0	0	0	0	9
	Median (IQR) ^12^	Calories (kcal)	132 (6)	-	-	127 (71)	132 (6)	48 (1)	-	-	-	-	127 (71)
		Sodium (mg)	123 (8)	-	-	317 (79)	123 (7)	159 (9)	-	-	-	-	317 (79)
		Saturated fat (g)	3 (0)	-	-	2 (3)	3 (1)	0 (0)	-	-	-	-	2 (3)
		Total sugars (g) ^13^	0 (0)	-	-	0 (0)	0 (0)	0 (0)	-	-	-	-	0 (0)
		Free sugars (g)	0 (0)			0 (0)	0 (0)	0 (0)	-	-	-	-	0 (0)
		Fibre (g)	0 (0)	-	-	2 (2)	0 (0)	0 (0)	-	-	-	-	2 (2)
		Protein (g)	11 (1)	-	-	11 (1)	11 (1)	11 (0)	-	-	-	-	11 (1)
H. Fats and oils	*n*		0	231	0	425	2	191	0	37	0	426	0
	Median (IQR) ^12^	Calories (kcal)	-	800 (0)	-	367 (300)	900 (0)	800 (0)	-	700 (100)	-	381 (300)	-
		Sodium (mg)	-	0 (0)	-	733 (300)	0 (0)	0 (0)	-	0 (700)	-	733 (296)	-
		Saturated fat (g)	-	15 (33)	-	3 (5)	60 (0)	15 (10)	-	35 (35)	-	3 (5)	-
		Total sugars (g) ^13^	-	0 (0)	-	7 (7)	0 (0)	0 (0)	-	0 (0)	-	7 (7)	-
		Free sugars (g)	-	0 (0)	-	6 (7)	0 (0)	0 (0)	-	0 (0)	-	6 (7)	-
		Fibre (g)	-	0 (0)	-	0 (0)	0 (0)	0 (0)	-	0 (0)	-	0 (0)	-
		Protein (g)	-	0 (0)	-	1 (1)	0 (0)	0 (0)	-	0 (1)	-	1 (1)	-
I. Marine and fresh water animals	*n*		57	0	202	187	57	0	13	189	0	0	187
	Median (IQR) ^12^	Calories (kcal)	82 (33)	-	144 (90)	164 (110)	82 (33)	-	123 (34)	145 (96)	-	-	164 (110)
		Sodium (mg)	124 (143)	-	372 (249)	400 (190)	124 (143)	-	80 (41)	382 (261)	-	-	400 (185)
		Saturated fat (g)	0 (1)	-	1 (2)	1 (1)	0 (1)	-	1 (1)	1 (2)	-	-	1 (1)
		Total sugars (g) ^13^	0 (0)	-	0 (0)	2 (3)	0 (0)	-	0 (0)	0 (0)	-	-	2 (3)
		Free sugars (g)	0 (0)	-	0 (0)	2 (3)	0 (0)	-	0 (0)	0 (0)	-	-	2 (3)
		Fibre (g)	0 (0)	-	0 (0)	1 (1)	0 (0)	-	0 (0)	0 (0)	-	-	1 (1)
		Protein (g)	18 (4)	-	21 (6)	12 (6)	18 (4)	-	24 (6)	20 (6)	-	-	12 (6)
J. Fruit and fruit juices	*n*		543	0	213	300	155	138	293	196	0	0	274
	Median (IQR) ^12^	Calories (kcal)	48 (13)	-	72 (265)	48 (12)	57 (226)	48 (4)	48 (10)	80 (261)	-	-	48 (13)
		Sodium (mg)	4 (9)	-	5 (18)	6 (10)	0 (8)	4 (8)	6 (8)	5 (24)	-	-	6 (10)
		Saturated fat (g)	0 (0)	-	0 (0)	0 (0)	0 (0)	0 (0)	0 (0)	0 (0)	-	-	0 (0)
		Total sugars (g) ^13^	10 (3)	-	15 (50)	11 (4)	10 (30)	9 (2)	10 (3)	16 (52)	-	-	11 (4)
		Free sugars (g)	8 (10)	-	8 (13)	10 (4)	0 (0)	9 (2)	10 (5)	9 (14)	-	-	10 (4)
		Fibre (g)	0 (2)	-	1 (2)	0 (0)	3 (5)	0 (0)	0 (1)	2 (3)	-	-	0 (0)
		Protein (g)	0 (1)	-	1 (1)	0 (0)	1 (2)	0 (1)	0 (1)	0 (1)	-	-	0 (0)
K. Legumes	*n*		92	0	91	5	79	0	32	72	0	0	5
	Median (IQR) ^12^	Calories (kcal)	337 (217)	-	80 (24)	73 (0)	340 (45)	-	88 (45)	80 (22)	-	-	73 (0)
		Sodium (mg)	5 (10)	-	96 (153)	3 (0)	5 (10)	-	4 (12)	110 (137)	-	-	3 (0)
		Saturated fat (g)	0 (0)	-	0 (0)	0 (0)	0 (0)	-	0 (1)	0 (0)	-	-	0 (0)
		Total sugars (g) ^13^	2 (1)	-	0 (1)	11 (1)	2 (1)	-	0 (0)	0 (1)	-	-	11 (1)
		Free sugars (g)	0 (0)	-	0 (0)	11 (1)	0 (0)	-	0 (0)	0 (0)	-	-	11 (1)
		Fibre (g)	16 (16)	-	4 (2)	0 (0)	17 (11)	-	3 (3)	4 (2)	-	-	0 (0)
		Protein (g)	22 (10)	-	5 (1)	3 (0)	22 (5)	-	6 (5)	5 (1)	-	-	3 (0)
L. Meat, poultry, their products and substitutes	*n*		31	0	93	836	30	0	0	130	0	0	800
	Median (IQR) ^12^	Calories (kcal)	190 (91)	-	240 (245)	214 (120)	199 (91)	-	-	242 (209)	-	-	212 (123)
		Sodium (mg)	60 (16)	-	635 (445)	653 (442)	60 (15)	-	-	457 (423)	-	-	676 (428)
		Saturated fat (g)	4 (4)	-	6 (11)	3 (7)	5 (4)	-	-	7 (8)	-	-	3 (7)
		Total sugars (g) ^13^	0 (0)	-	0 (0)	1 (3)	0 (0)	-	-	0 (0)	-	-	1 (3)
		Free sugars (g)	0 (0)	-	0 (0)	0 (2)	0 (0)	-	-	0 (0)	-	-	1 (2)
		Fibre (g)	0 (0)	-	0 (0)	0 (1)	0 (0)	-	-	0 (0)	-	-	0 (1)
		Protein (g)	19 (3)	-	18 (9)	16 (7)	19 (2)	-	-	17 (6)	-	-	16 (7)
M. Miscellaneous category ^14^	*n*		29	101	0	420	11	53	0	74	0	197	215
	Median (IQR) ^12^	Calories (kcal)	117 (121)	200 (300)	-	308 (151)	0 (250)	167 (400)	-	200 (300)	-	333 (161)	295 (149)
		Sodium (mg)	12 (19)	9500 (15,867)	-	398 (2731)	0 (0)	27 (7488)	-	9500 (12,395)	-	3333 (6600)	336 (133)
		Saturated fat (g)	5 (13)	0 (0)	-	1 (3)	0 (0)	5 (14)	-	0 (0)	-	0 (0)	3 (4)
		Total sugars (g) ^13^	0 (2)	0 (0)	-	14 (26)	0 (0)	0 (2)	-	0 (0)	-	3 (17)	20 (22)
		Free sugars (g)	0 (0)	0 (0)	-	12 (25)	0 (0)	0 (0)	-	0 (0)	-	0 (16)	20 (26)
		Fibre (g)	0 (0)	0 (0)	-	1 (3)	0 (10)	0 (7)	-	0 (0)	-	0 (3)	1 (3)
		Protein (g)	1 (2)	3 (11)	-	5 (7)	10 (20)	1 (8)	-	1 (10)	-	8 (8)	4 (2)
N. Combination dishes	*n*		0	0	0	1130	0	0	0	0	0	0	1130
	Median (IQR) ^12^	Calories (kcal)	-	-	-	163 (112)	-	-	-	-	-	-	163 (112)
		Sodium (mg)	-	-	-	333 (198)	-	-	-	-	-	-	333 (198)
		Saturated fat (g)	-	-	-	2 (3)	-	-	-	-	-	-	2 (3)
		Total sugars (g) ^13^	-	-	-	2 (2)	-	-	-	-	-	-	2 (2)
		Free sugars (g)	-	-	-	1 (3)	-	-	-	-	-	-	1 (3)
		Fibre (g)	-	-	-	1 (1)	-	-	-	-	-	-	1 (1)
		Protein (g)	-	-	-	7 (4)	-	-	-	-	-	-	7 (4)
O. Nuts and seeds	*n*		148	48	0	59	143	0	43	48	0	0	21
	Median (IQR) ^12^	Calories (kcal)	650 (117)	625 (36)	-	588 (69)	650 (100)	-	625 (43)	600 (48)	-	-	531 (143)
		Sodium (mg)	0 (8)	0 (33)	-	300 (151)	0 (8)	-	0 (31)	306 (116)	-	-	109 (218)
		Saturated fat (g)	6 (4)	7 (1)	-	8 (4)	6 (4)	-	7 (2)	8 (3)	-	-	6 (4)
		Total sugars (g) ^13^	3 (2)	6 (4)	-	11 (14)	3 (2)	-	7 (2)	7 (3)	-	-	25 (21)
		Free sugars (g)	0 (0)	0 (0)	-	7 (16)	0 (0)	-	0 (0)	2 (5)	-	-	23 (23)
		Fibre (g)	8 (5)	7 (0)	-	7 (1)	8 (5)	-	7 (3)	7 (0)	-	-	6 (3)
		Protein (g)	20 (8)	25 (8)	-	19 (6)	20 (7)	-	25 (7)	20 (5)	-	-	15 (6)
P. Potatoes, sweet potatoes and yams	*n*		14	0	15	103	14	0	2	40	0	0	76
	Median (IQR) ^12^	Calories (kcal)	68 (13)	-	56 (36)	141 (59)	68 (13)	-	120 (0)	129 (54)	-	-	153 (79)
		Sodium (mg)	12 (17)	-	112 (96)	290 (187)	12 (17)	-	75 (0)	112 (124)	-	-	327 (124)
		Saturated fat (g)	0 (0)	-	0 (0)	1 (1)	0 (0)	-	0 (0)	0 (0)	-	-	1 (1)
		Total sugars (g) ^13^	1 (1)	-	0 (6)	0 (1)	1 (1)	-	22 (0)	0 (0)	-	-	1 (2)
		Free sugars (g)	0 (0)	-	0 (0)	0 (0)	0 (0)	-	0 (0)	0 (0)	-	-	0 (1)
		Fibre (g)	2 (1)	-	2 (1)	2 (1)	2 (1)	-	2 (0)	2 (1)	-	-	2 (1)
		Protein (g)	2 (0)	-	1 (1)	2 (0)	2 (0)	-	1 (0)	2 (1)	-	-	2 (1)
Q. Salads	*n*		2	0	14	113	2	0	0	1	0	0	126
	Median (IQR) ^12^	Calories (kcal)	42 (18)	-	127 (27)	141 (84)	42 (18)	-	-	106 (0)	-	-	140 (81)
		Sodium (mg)	61 (51)	-	310 (167)	270 (213)	61 (51)	-	-	3 (0)	-	-	274 (213)
		Saturated fat (g)	0 (0)	-	1 (1)	2 (1)	0 (0)	-	-	0 (0)	-	-	2 (1)
		Total sugars (g) ^13^	1 (0)	-	4 (6)	3 (3)	1 (0)	-	-	8 (0)	-	-	3 (3)
		Free sugars (g)	0 (0)	-	0 (5)	1 (4)	0 (0)	-	-	6 (0)	-	-	1 (4)
		Fibre (g)	2 (0)	-	2 (3)	2 (1)	2 (0)	-	-	2 (0)	-	-	2 (1)
		Protein (g)	3 (0)	-	4 (4)	4 (4)	3 (0)	-	-	2 (0)	-	-	4 (4)
R. Sauces, dips, gravies and condiments	*n*		0	36	0	1203	0	0	0	0	0	1239	0
	Median (IQR) ^12^	Calories (kcal)	-	100 (167)	-	117 (144)	-	-	-	-	-	117 (144)	-
		Sodium (mg)	-	0 (5)	-	533 (758)	-	-	-	-	-	517 (750)	-
		Saturated fat (g)	-	0 (0)	-	0 (2)	-	-	-	-	-	0 (1)	-
		Total sugars (g) ^13^	-	20 (33)	-	5 (18)	-	-	-	-	-	6 (18)	-
		Free sugars (g)	-	0 (0)	-	2 (19)	-	-	-	-	-	2 (16)	-
		Fibre (g)	-	0 (0)	-	0 (2)	-	-	-	-	-	0 (2)	-
		Protein (g)	-	0 (1)	-	2 (3)	-	-	-	-	-	2 (3)	-
S. Snacks	*n*		10	0	132	719	10	0	0	359	0	0	492
	Median (IQR) ^12^	Calories (kcal)	380 (97)	-	633 (60)	500 (80)	380 (97)	-	-	540 (125)	-	-	500 (69)
		Sodium (mg)	5 (13)	-	185 (308)	560 (423)	5 (13)	-	-	340 (401)	-	-	620 (427)
		Saturated fat (g)	1 (3)	-	8 (4)	3 (4)	1 (3)	-	-	5 (6)	-	-	3 (4)
		Total sugars (g) ^13^	0 (19)	-	6 (2)	4 (6)	0 (19)	-	-	4 (6)	-	-	4 (4)
		Free sugars (g)	0 (0)	-	0 (0)	1 (3)	0 (0)	-	-	0 (0)	-	-	2 (5)
		Fibre (g)	14 (6)	-	7 (4)	4 (4)	14 (6)	-	-	7 (5)	-	-	4 (2)
		Protein (g)	10 (2)	-	20 (4)	8 (6)	10 (2)	-	-	10 (3)	-	-	7 (7)
T. Soups	*n*		0	0	0	480	0	0	0	0	0	0	480
	Median (IQR) ^12^	Calories (kcal)	-	-	-	40 (54)	-	-	-	-	-	-	40 (54)
		Sodium (mg)	-	-	-	260 (101)	-	-	-	-	-	-	260 (101)
		Saturated fat (g)	-	-	-	0 (1)	-	-	-	-	-	-	0 (1)
		Total sugars (g) ^13^	-	-	-	1 (1)	-	-	-	-	-	-	1 (1)
		Free sugars (g)	-	-	-	0 (1)	-	-	-	-	-	-	0 (1)
		Fibre (g)	-	-	-	0 (1)	-	-	-	-	-	-	0 (1)
		Protein (g)	-	-	-	1 (1)	-	-	-	-	-	-	1 (1)
U. Sugars and sweets	*n*		6	117	0	983	43	69	0	179	0	219	596
	Median (IQR) ^12^	Calories (kcal)	300 (0)	350 (75)	-	389 (199)	300 (0)	375 (25)	-	300 (133)	-	333 (267)	477 (189)
		Sodium (mg)	0 (0)	0 (2)	-	38 (90)	0 (0)	0 (2)	-	0 (27)	-	7 (73)	66 (90)
		Saturated fat (g)	0 (0)	0 (0)	-	1 (17)	0 (0)	0 (0)	-	0 (0)	-	0 (11)	13 (20)
		Total sugars (g) ^13^	80 (0)	83 (20)	-	52 (21)	80 (0)	100 (17)	-	60 (20)	-	53 (37)	49 (16)
		Free sugars (g)	80 (0)	83 (20)	-	48 (23)	80 (0)	100 (17)	-	43 (25)	-	50 (43)	48 (18)
		Fibre (g)	0 (0)	0 (0)	-	0 (3)	0 (0)	0 (0)	-	0 (0)	-	0 (0)	2 (5)
		Protein (g)	0 (0)	0 (0)	-	3 (7)	0 (0)	0 (0)	-	1 (1)	-	0 (5)	5 (5)
V. Vegetables	*n*		233	0	491	142	213	26	58	434	0	15	120
	Median (IQR) ^12^	Calories (kcal)	35 (21)	-	32 (63)	28 (83)	35 (23)	28 (18)	26 (28)	33 (80)	-	100 (17)	24 (59)
		Sodium (mg)	24 (41)	-	240 (832)	500 (649)	24 (43)	38 (47)	8 (13)	296 (868)	-	633 (400)	515 (652)
		Saturated fat (g)	0 (0)	-	0 (0)	0 (0)	0 (0)	0 (0)	0 (0)	0 (0)	-	0 (0)	0 (0)
		Total sugars (g) ^13^	2 (2)	-	2 (3)	3 (13)	2 (2)	4 (4)	2 (2)	2 (3)	-	20 (3)	3 (9)
		Free sugars (g)	0 (0)	-	0 (0)	0 (12)	0 (0)	0 (0)	0 (0)	0 (0)	-	19 (4)	0 (9)
		Fibre (g)	2 (2)	-	2 (2)	0 (1)	2 (2)	1 (1)	2 (1)	2 (3)	-	0 (0)	0 (1)
		Protein (g)	2 (2)	-	1 (1)	1 (1)	2 (2)	1 (1)	1 (1)	1 (2)	-	1 (0)	0 (1)

^1^ Food categories are defined in Health Canada’s Table of Reference Amounts for Food [37]. ^2^ U/MP: unprocessed or minimally-processed products; ^3^ PCI: processed culinary ingredients. ^4^ P: processed foods. ^5^ UP: ultra-processed food and drink products. ^6^ BPI: basic processed ingredients. ^7^ PBP: processed for basic preservation. ^8^ MPF: moderately processed for flavour. ^9^ MPG: moderately processed grain products. ^10^ HPI: highly processed ingredients. ^11^ HPS: highly processed stand-alone. ^12^ IQR: interquartile range. ^13^ Medians of free sugars exclude products without an ingredients list as free sugars could not be estimated (*n* = 433). Free sugars were estimated for the remaining 16,836 products with an ingredients list. ^14^ Examples of products in the Miscellaneous category: baking powder, baking decorations, bread crumbs, batter mixes, cocoa powder, salad toppers, salt, spices and herbs, coconut milk, etc.

## Supplementary Materials

The following are available online at https://www.mdpi.com/2072-6643/11/11/2782/s1, Table S1: Definitions and example foods for each category of the NOVA and Poti et al. food processing classification systems.
