# Supplementary material for: The Calorie and Nutrient Density of More- Versus Less-Processed Packaged Food and Beverage Products in the Canadian Food Supply
Source: Nutrients. 2019 Nov 15;11(11):2782. doi: 10.3390/nu11112782 (PMC6893502; doi:10.3390/nu11112782)
Supplement: Supplementary file 1 [file nutrients-11-02782-s001.pdf]

**Table S1.** Definitions and example foods for each category of the NOVA [1] and Poti et al. [2] food processing classification systems.

| Classification system | Category | Definition | Examples of foods |
|---|---|---|---|
| NOVA | Unprocessed or minimally processed foods | 'Fresh' or 'whole' foods derived from plants or animals without any industrial processing; foods that are processed or altered to improve their palatability and shelf-life, or facilitate their cooking and preparation, without the addition of fats, sugars or salt | Fruits and vegetables (fresh, dry or frozen); grains; legumes; nuts, meats; seafood; eggs; unflavoured milk; plain yogurt; tea, coffee, drinking water; dried mixed fruits with no added sugar; granola made from cereals, nuts and dried fruits without added sugars or oil |
| Poti et al. | Unprocessed/minimally processed | Foods with a single ingredient that have undergone no or minimal alterations; inherent properties of the food in its natural form remain unchanged | Fresh plain milk, coffee; tea bags or leaves; bottled plain water; plain fruits, vegetables or legumes (fresh, frozen or dried); plain nuts; eggs; whole-grain hot cereal; brown rice; cream; honey; herbs and spices |
| NOVA | Processed culinary ingredients | Ingredients extracted and refined from food components or derived from nature, typically not consumed in isolation but rather, are used in the preparation and cooking of foods to increase their palatability and diversity | Salt; sugar; molasses; honey; maple syrup; vegetable oils; butter (unsalted or salted); lard; corn starch and other plant starches |
| Poti et al. | Basic processed | | |
| | Processed basic ingredients | Single ingredients obtained using physical or chemical processes to extract or purify a food component; inherent properties of the food are altered | Unsweetened fruit juice not from concentrate; egg whites; whole-grain flour or pasta; oil; unsalted butter; sugar; pure maple syrup; salt |
| | Processed for basic preservation | Foods that have been minimally processed for preservation or | Unsweetened fruit juice from concentrate; powdered milk; instant coffee; unsweetened/unflavoured canned |

| **Classification system** | **Category** | **Definition** | **Examples of foods** |
|---|---|---|---|
| | | precooking purposes; remains as a single food, but inherent properties are altered | fruit, vegetables or legumes; nut butters without added salt or sugar; unseasoned canned meat; refined-grain pasta or flour; white and/or instant rice; plain yogurt |
| NOVA | Processed foods | Foods produced by adding fats, oils, sugars, salt and other culinary ingredients to increase their durability and palatability, usually through some form of preservation | Meats that have been salted, pickled or cured; canned fish; vegetables, fruits, legumes or animal products preserved in oil, syrup or brine; unpackaged freshly-made breads and cheeses |
| Poti et al. | Moderately processed | | |
| | Moderately processed for flavour | Minimally or moderately processed food containing flavour additives (e.g., sugar) for improving palatability; food is still recognizable as its original source | Sweetened/flavoured juice or milk; sweetened/flavoured canned, dried, refrigerated or frozen fruit, vegetables or legumes; potato chips; frozen French fries; salted nut butter; sweetened/flavoured hot cereal or yogurt; cheese; salted butter; flavoured pasta or oil |
| | Moderately processed grain products | Grain products made from whole-grain flour, water, salt and/or yeast (i.e., no added sugar, fat or modified ingredients) | Whole-grain breads, tortillas, crackers or ready-to-eat cereals with no added sugar or fat |
| NOVA | Ultra-processed food and drink products | Products that are primarily formulations of industrial ingredients and other substances derived from foods (e.g., fats, oils, starches, sugar), and additives such as preservatives, antioxidants and stabilizers; typically contain minimal amounts of intact food, or none at all | Carbonated beverages; sweet or savoury packaged snacks; ice-cream; chocolate; confectionary; mass-produced packaged breads and buns; breakfast cereals and cereal or energy bars; sweetened yogurts; instant sauces; pre-prepared meals (e.g., pasta dishes, pizza); reconstituted meat products (e.g., breaded nuggets, hot dogs, burgers); instant soups, noodles and desserts |

| Classification system | Category | Definition | Examples of foods |
|---|---|---|---|
| Poti et al. | Highly processed | | |
| | Highly processed ingredients | Mixtures of multiple ingredients that have been industrially-formulated and processed to such a degree that they are not recognizable as their original source; consumed as additions to foods (e.g., sauces, dips, toppings) | Tomato sauce, salsa, hummus, bread crumbs with refined grains or added sugar/fat; creamer, dips, margarine, artificial sweeteners; instant sauces, condiments and seasoning mixes |
| | Highly processed stand-alone | Mixtures of multiple ingredients that have been industrially-formulated and processed to such a degree that they are not recognizable as their original source; not usually consumed as additions to other foods (i.e., other ingredients not required) | Carbonated beverages; flavoured waters; fruit snacks; frozen vegetables in sauce; restructured potato chips; reformed French fries; sausage; hot dogs; lunch meats; breaded meats; bread products with refined grains and/or added sugar or fat; processed cheese; pre-prepared/instant meals; broth; ice cream and other pre-prepared desserts; confectionary |

[1]Monteiro, C.A.; Cannon, G.; Levy, R.; Moubarac, J.-C.; Jaime, P.; Martins, A.P.; Canella, D.; Louzada, M.; Parra, D. NOVA. The star shines bright. *World Nutrition* **2016**, *7*, 28-38. [2]Poti, J.M.; Mendez, M.A.; Ng, S.W.; Popkin, B.M. Is the degree of food processing and convenience linked with the nutritional quality of foods purchased by US households? *Am J Clin Nutr* **2015**, *101*, 1251-1262, doi:10.3945/ajcn.114.100925.
